# Paclitaxel induces cognitive impairment via necroptosis, decreased synaptic plasticity and M1 polarisation of microglia

**DOI:** 10.1080/13880209.2022.2108064

**Published:** 2022-08-09

**Authors:** Miao Tang, Shuang Zhao, Jia-Xin Liu, Xin Liu, Yue-Xian Guo, Gui-Ying Wang, Xiu-Li Wang

**Affiliations:** a Department of Anesthesiology, The Third Hospital of HeBei Medical University, Shijiazhuang, China; b Department of Surgery, the Third Affiliated Hospital of Hebei Medical University, Shijiazhuang, China

**Keywords:** Central neurotoxicity, hippocampus, neuron, astrocytes, inflammatory factors

## Abstract

**Context:**

Paclitaxel (PTX) leads to chemotherapy brain (chemo-brain) which is characterised by cognitive impairment. It has been reported that necroptosis is associated with cognitive impairment in some neurodegenerative diseases, but it is not clear whether it is related to the development of chemo-brain.

**Objective:**

To investigate the role of necroptosis and related changes in PTX-induced cognitive impairment.

**Materials and methods:**

C57bl/6n mice were randomly divided into five groups: control, vehicle, and different concentrations of PTX (6, 8, 10 mg/kg). Two additional groups received pre-treatment with Gdcl3 or PBS through Intracerebroventricular (ICV) injection before PTX-treatment. Cognitive function, necroptosis, synaptic plasticity and microglia polarisation were analysed.

**Results:**

PTX (10 mg/kg) induced significant cognitive impairment, accompanied by changes in synaptic plasticity, including decreased density of PSD95 (0.65-fold), BDNF (0.44-fold) and dendritic spines (0.57-fold). PTX induced necroptosis of 53.41% (RIP3) and 61.91% (MLKL) in hippocampal neurons, with high expression of RIP3 (1.58-fold) compared with the control group. MLKL (1.87-fold) exhibited the same trend, reaching a peak on the 14th day. The increased expression of iNOS (1.63-fold) and inflammatory factors such as TNF-α (1.85-fold) and IL-β (1.89-fold) compared to the control group suggests that M1 polarisation of microglia is involved in the process of cognitive impairment. Pre-treatment with Gdcl3 effectively reduced the number of microglia (0.50-fold), inhibited the release of TNF-α (0.73-fold) and IL-β (0.56-fold), and improved cognitive impairment.

**Conclusion:**

We established a stable animal model of PTX-induced cognitive impairment and explored the underlying pathophysiological mechanism. These findings can guide the future treatment of chemo-brain.

## Introduction

The central neurotoxicity of paclitaxel (PTX) has received increasing attention in recent years. PTX-induced central neurotoxicity is characterised by cognitive disorder and acute encephalopathy, with different degrees and intensities of emotional disturbances (Boyette-Davis and Fuchs 2009). 15–75% of breast cancer patients have mild to moderate cognitive dysfunction after receiving PTX chemotherapy, with 35% of patients experiencing symptoms that persist for years (Schagen et al. 1999; Tannock et al. 2004). Despite description of the central neurotoxicity caused by PTX from a clinical perspective, there is little understanding of the specific pathological mechanisms. Studies have found that PTX can enter the brain through the blood-brain barrier (BBB), causing dose-dependent neurotoxicity and neuronal apoptosis (Karavelioglu et al. 2016). We have reason to believe that PTX-induced cognitive impairment may be related to the destruction of the structure and function of the hippocampus, which is the cognitive centre of the brain.

Necroptosis is a special form of cell necrosis that has recently been discovered. It is strictly regulated by receptor-interacting protein-3 (RIP3) and mixed lineage kinase domain-like protein (MLKL). Studies have shown that it is a controlled form of cell necrosis with necrotic characteristics, including cell swelling and rupture of the cell membrane, accompanied by the release of inflammatory factors (He et al. 2009; Linkermann and Green 2014; Mifflin et al. 2020). Damage-associated molecular patterns (DAMPs) and the release of inflammatory factors are involved in the occurrence of many degenerative diseases of the central nervous system, including Alzheimer’s disease (AD), Parkinson’s disease (PD) and amyotrophic lateral sclerosis (ALS) (Re et al. 2014; Fischer and Maier 2015; Han et al. 2016). Several studies have confirmed that PTX can induce apoptosis of hippocampal neurons, accompanied by the release of inflammatory factors such as TNF-α and IL-1 β (Li et al. 2018). However, the specific mechanism requires further study.

Synaptic formation, elimination and synaptic plasticity contribute to cognitive function, including learning, memory, emotion and behaviour (Malenka and Bear 2004; Neves et al. 2008; Jang and Chung 2016). Drug interventions can improve the cognitive impairment observed in AD by improving synaptic plasticity in the hippocampus. Researchers have also observed a decrease in brain-derived neurotrophic factor (BDNF) and synaptic proteins in the hippocampus, including synaptophysin (SYN) and postsynaptic density protein 95 (PSD95), in stress-induced depression-like behaviour and cognitive impairment (Shen et al. 2019). Therefore, damage to the synaptic structure and a decrease in synaptic plasticity induced by PTX may be the mechanism underlying cognitive impairment.

Microglia are innate immune components of the central nervous system that play a similar function to peripheral macrophages. The phenotype of microglia can be changed based on changes in the surrounding environment and stimulation signals; activated microglia may be in a pro-inflammatory state (M1) or anti-inflammatory state (M2), with each state corresponding to specific inflammatory factors (Li and Barres 2018; Park et al. 2021). We observed the release of a large number of inflammatory factors in the hippocampus of mice treated with PTX in our previous study (Li et al. 2018). Therefore, we speculate that the polarisation of microglia is also involved in the process of necroptosis, but the specific mechanism is not yet clear. As a scavenger, gadolinium chloride (Gdcl3) can non-selectively reduce the number of microglia, thereby inhibiting the inflammatory reaction. Based on these findings, we hypothesise that the neurotoxicity caused by the overactivation of microglia and the release of inflammatory factors may underlie the cognitive impairment induced by PTX.

To this end, this study explores the molecular mechanism of PTX-induced cognitive impairment. We speculate that the occurrence of necroptosis of hippocampal cells may be a key factor in PTX-induced cognitive injury and that synaptic plasticity damage and M1 polarisation of microglia are also involved in this process.

## Materials and methods

### Animals

All experimental protocols and animal handling procedures were performed in accordance with the National Institute of Health guidelines and regulations. The experimental protocols were approved by the Experimental Animal Welfare Ethics Committee of Hebei Medical University (Approval No. IACUC-Hebmu-2020009). Six to eight-week-old male C57bl/6n mice (weighing 20–25 g at the start of the experiment) were provided by the Beijing Vital River Laboratory Animal Technology Co., Ltd. (Beijing, China). Five animals were housed per cage under standard laboratory conditions.

**Figure 1.**
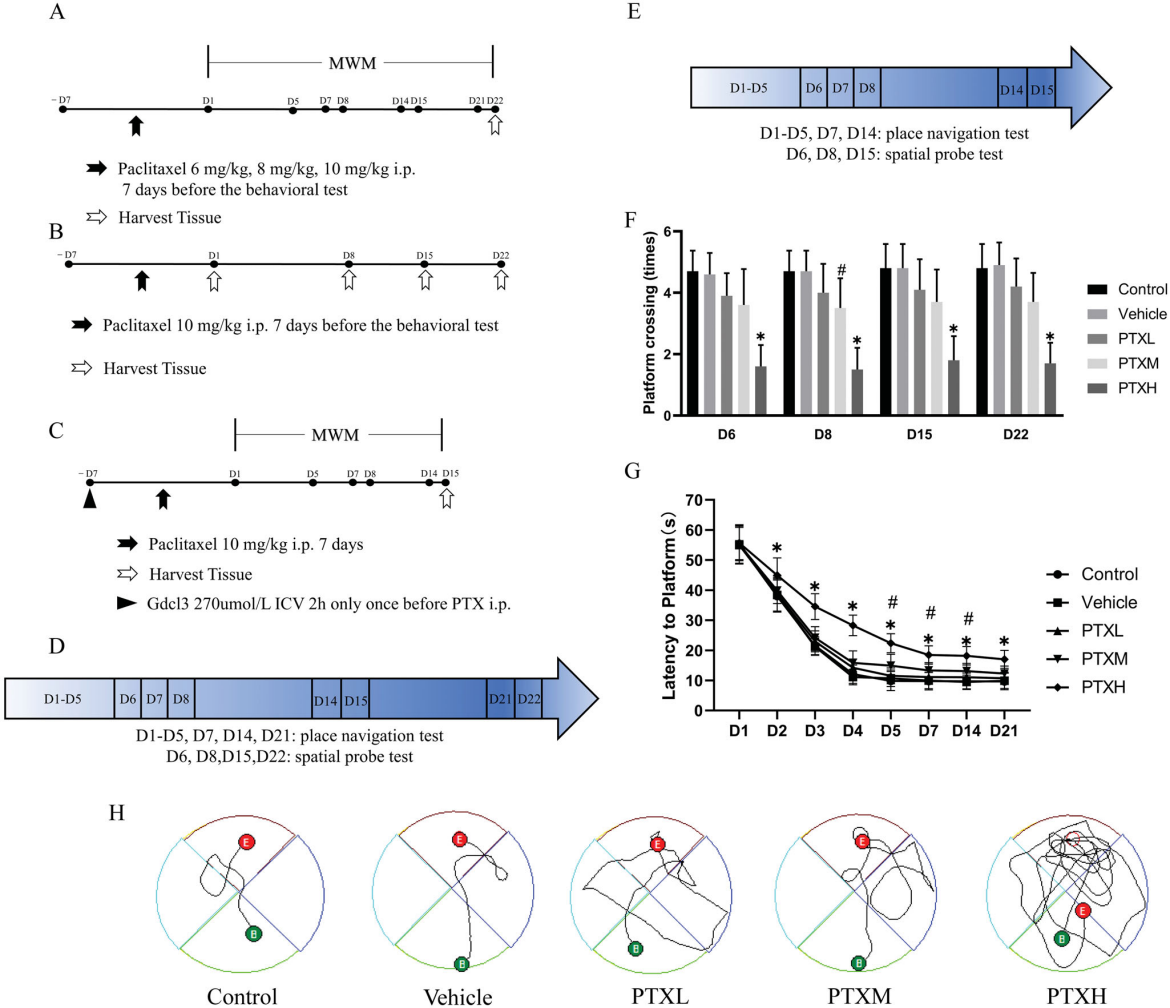
The behavioural test of PTX-treated mice under the different experimental conditions. (A, B, C) Experimental protocol. (D, E) Morris water maze flowchart. (F) The escape latency(s). (G) The number of times the mice passed over the platform. (H) Movement trajectories of mice on 21st day. The results are presented as the mean ± *SD*, *n* = 10. In the PTXM group, ^#^*p* < 0.05 vs. vehicle. In the PTXH group, *p < 0.05 vs. vehicle.

As shown in Figure 1(A), the experiment was divided into two parts. First of all, the adult male mice (*n* = 50) were randomly assigned to five groups: (1) control; mice in the control group did not undergo any experimental procedures; (2) vehicle; mice in this group received PTX dissolved in anhydrous ethanol and Cremophor EL (1:1); (3) PTXL; mice in this group were injected with PTX 6 mg/kg; (4) PTXM; mice in this group were injected with PTX 8 mg/kg; (5) PTXH; mice in this group were injected with PTX 10 mg/kg, all the mice received seven intraperitoneal injections before the behaviour test began. After the completion of this part of experiment, the other mice received intraperitoneal injection of PTX 10 mg/kg and the hippocampal tissues were collected at each time point as shown in Figure 1(B) (*n* = 12). For the study of microglia, as shown in Figure 1(C), the mice (*n* = 20) were randomly divided into two groups: (1) PTXH + PBS; mice in this group received intracerebroventricular injection of PBS solution before PTX treatment; (2) PTXH + Gdcl3; mice in this group received intracerebroventricular injection of Gdcl3 solution 2 h before PTX treatment only once.

### Drugs and treatment

PTX was dissolved in a derivative of castor oil and ethylene oxide mixed with anhydrous alcohol before injection. PTX was diluted to 0.4 mg/mL of working solution before use. After habituation to the test environment and baseline measurement of behavioural tests, different concentrations of PTX or vehicle were administered to the relevant groups of mice for seven consecutive days. Gdcl3 was dissolved in PBS and then prepared to a concentration of 270 mmol/L and a volume of 1 mL; it was then diluted to 270 μmol/L for use.

### Intracerebroventricular (ICV) injection

Drug administration into the intracerebral ventricle was performed as previously described by Layé et al. (2000). After the mouse was anaesthetised by pentobarbital (50 mg/kg), its head was fixed in a stereotaxic frame. The position of the right cerebral ventricle was defined by the following coordinates: 0.6 mm posterior and 1.2 mm lateral to the bregma, and 1.8 mm below the skull surface. Then, 5 μL of the drug was injected at a rate of 0.5 μL/min.

### The Morris Water Maze (MWM)

The MWM test was used to evaluate the spatial learning and memory functions of mice. All mice were processed for 5 days before the MWM test. The platform was placed above the water surface (visible platform) for memory training for 5 days; four tests were performed per day (each experiment lasted up to 60 s, and the interval between each experiment was greater than 20 min). Each mouse was placed in the tank in one of four different directions, and the latency to reach the platform was recorded. The time from the opposite side of the platform to the platform was calculated as the escape latency. On the 6th day, the platform was removed for the probe trial. Experiment with spatial exploration in the second 24 h. The escape latency and the number of times each mouse crossed the platform were tested in the 1st, 2nd and 3rd weeks following PTX treatment. The behaviour test schedules are illustrated in Figure 1(D,E).

### Western blot

Western blot was performed as Li et al. (2018) reported. Hippocampal tissues were harvested after the behavioural tests and homogenised in RIPA lysis buffer containing protease inhibitor cocktail (Abcam, ab65621). An equal amount of protein (40 µg) was loaded into each lane and separated by SDS-PAGE then transferred to PVDF membranes (Millipore USA) by electrophoresis. The membranes were blocked with 5% non-fat skim milk in TBST (0.1% Tween 20 in TBS) for 1 h at room temperature and then incubated overnight at 4 °C with anti-RIP3 (1:1000, Enzo, ADI-905-242), anti-MLKL (1:1000, Millipore, MABC604) and anti-β-actin (1:5000, ABclonal, AC206). The membranes were washed three times with TBST and then incubated with goat anti-rabbit infra-red fluorescent antibody (1:10,000, Rockland, 611-145-002) for 2 h at room temperature. An Odyssey 9120 infra-red imaging system (LI-COR, USA) and ImageJ software were used to quantify the protein bands. All experiments were repeated in triplicate.

### Immunofluorescence

The expression of RIP3, MLKL, NeuN, GFAP, Iba-1, Arg-1 and iNOS was used to observe the necroptosis of hippocampal cells and the polarisation of microglia. After the brain tissues were removed, they were fixed with 4% paraformaldehyde and gradient dehydration was performed with sucrose solution. Then, the tissues were embedded in OCT. The specimens were cut into 10 μm sections with a microtome. After antigen retrieval, the sections were blocked with 10% donkey serum for 30 min. Then, they were incubated with primary antibodies against RIP3, MLKL, NeuN (1:200, Arigo, ARG52283), GFAP (1:1000, Arigo, ARG52312), Iba-1 (1:200, GeneTex, GTX632426), Arg-1 (1:500, BD, 610708) and iNOS (1:200, Arigo, ARG56509) at 4 °C overnight. Subsequently, the sections were processed with the secondary antibody at room temperature for 50 min in the dark. Then, DAPI solution was used to counterstain the nuclei. Microscopy detection and image collection were performed by fluorescence microscopy. The nuclei were labelled blue with DAPI. The positive cells were green or red according to the fluorescent labels used (PANNORAMIC DESK/MIDI/250/1000).

### Enzyme-linked immunosorbent assay (ELISA)

The washed hippocampal tissues were added to a glass homogeniser and PBS was added at a weight ratio of 1:9. The homogenate was kept on ice. After the homogenate was mixed well, it was centrifuged for 15 min at 12,000 rpm. Then, the supernatant was removed for detection according to the specific steps for BDNF (ABclonal, RK00433), TNF-α (Invitrogen, 88-7013), IL-1β (Invitrogen, 88-7324), IL-4 (CUSABIO, CSB-E04634m) and IL-10 (MULTI SCIENCE, EK210/4-03). The optical density at 450 nm was determined by a multimode microplate reader (BioTeK, Epoch, USA).

### Golgi-Cox staining and dendritic spine counting

Golgi staining was used to observe the plasticity of synaptic structures. The mouse hippocampus tissues were immersed in Golgi staining solution (Servicebio, G1069) and stored in the dark for 2 weeks, according to the manufacturer’s instructions. Then, they were immersed in 80% glacial acetic acid overnight. When the tissues were soft, they were washed with distilled water and placed into 30% sucrose. Next, the tissues were cut into 100 µm sections with an oscillating microtome. A Nikon Eclipse E100 was used for imaging of the dendrites and dendritic spines in the hippocampus. A Pannoramic 250 and Nikon DS-U3 were used for scanning and analysis, respectively.

### Transmission electron microscopy

After the behavioural assays, the mice were anaesthetised with 1% sodium pentobarbital and sacrificed. The hippocampi were fixed in 2% paraformaldehyde plus 2% glutaraldehyde (pH 7.4) for 2 h and were then were sectioned (1 × 1 × 1 mm^3^). The post-fixed tissues were incubated in 1% osmium tetroxide (0.1 M, pH 7.4) and dehydrated in ascending concentrations of ethanol and acetone at room temperature. They were then embedded in resin. Ultrathin sections (60 nm) were obtained using an ultramicrotome (UC-7, Leica, Germany) and the sections were stained with lead citrate and uranyl acetate. The samples were detected using a transmission electron microscope (H-7500, Hitachi, Japan) for analysis at 6000× and 12,000× magnification.

### Immune electron microscopy

The immune electron microscopic study was performed as previously described. Briefly, 14 d after PTX treatment, the hippocampi were removed and cut to a size of 1 mm^3^. The tissues were then fixed with fresh IEM fixative (Servicebio, 1124-100ML) for 3 h. After dehydration with gradient alcohol and embedding in resin, tissue sections of 50 μm were prepared with an ultra-microtome. The sections were blocked with 5% BSA and 5% normal goat serum, incubated with anti-RIP3 or MLKL antibodies, and then with goat anti-rabbit IgG conjugated to 1.4 nm gold particles (1:100, Sigma, G7402) at room temperature overnight. After staining with 2% uranium acetate saturated alcohol solution, the sections were examined, and images were taken under a transmission electron microscope (Hitachi Tokyo). The 10 nm black golden particles were considered positive signals.

### Statistical analysis

Statistical analyses were performed using SPSS 26 (Chicago, IL). The data are presented as the mean ± *SD* and were analysed by one-way ANOVA followed by LSD *post hoc* tests. Behavioural results that were not normally distributed and/or had uneven variance were analysed with the Kruskal-Wallis H test. *p* Values less than 0.05 were considered statistically significant.

## Results

### PTX induced cognitive impairment in mice

The mice received different concentrations of PTX solution on days 1–7 before the MWM test began. The escape latency and the number of crossings at different time points were measured to determine the cognitive function of the mice. As shown in Figure 1(F,G), the mice treated with the middle dose of PTX (PTXM 8 mg/kg) had a significant decrease in the escape latency on days 5, 7 and 14 (*p* < 0.05). However, the results of the platform crossing test, which is another indicator of cognitive ability, revealed that the difference between the PTXM group and the vehicle group was only significant on day 8. The mice that received the high dose PTX (PTXH 10 mg/kg) treatment had an increased escape latency, and the number of crossings over the hidden escape platform was decreased compared with vehicle-treated mice, at all time points. Similarly, an examination of the representative swimming paths on the 22nd day showed that the degree of cognitive impairment in mice was positively correlated with the dose of PTX (Figure 1(H)). These data indicate that different concentrations of PTX can induce cognitive impairment in mice. Given the remarkable experimental effects, the high dose of PTX was used for the subsequent experimental procedures.

### PTX caused damage to synaptic structures and plasticity

**Figure 2.**
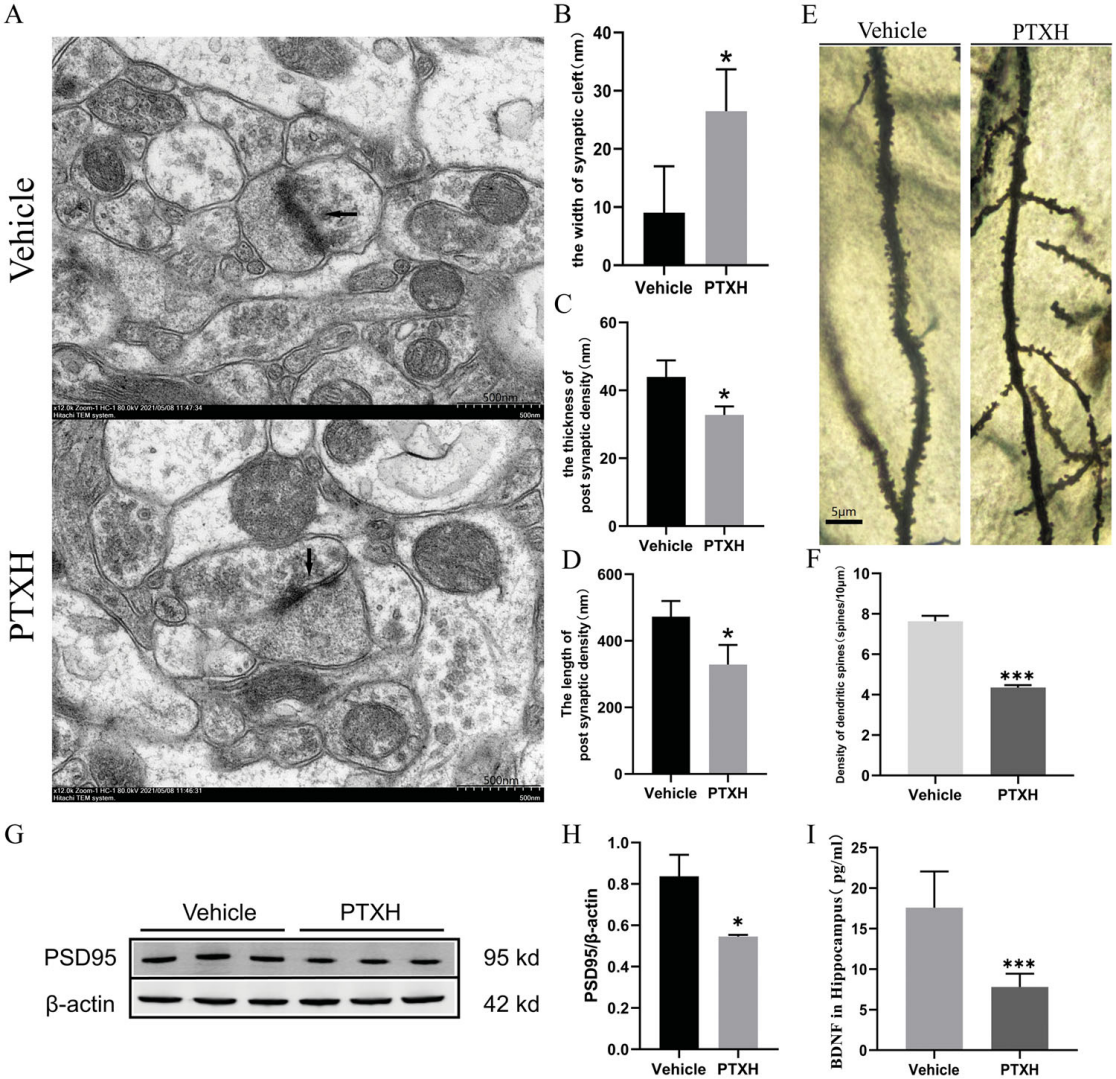
Effect of PTX on synaptic structures and plasticity. (A) Electron microscope images of synapses (scale bar = 500 nm). (B) The width of synaptic cleft. (C) The thickness of post synaptic density. (D) The length of post synaptic density. (E) Golgi staining of the hippocampus of mice. (F) The density of dendritic spines. (G, H) The expression of PSD95 in the hippocampus. (I) The concentration values of BDNF in the hippocampus. The results are presented as the mean ± *SD*, *n* = 3. In the PTXH group, *p < 0.05 vs. vehicle, **p < 0.01 vs. vehicle, ***p < 0.001 vs. vehicle.

Changes to synaptic structure and plasticity were directly related to cognitive impairment. The synapses of neurons were scanned by an electron microscope and in mice treated with PTXH, structural changes to the synapses, characterised by widening of the synaptic cleft, decreased length and thickness of post synaptic density, were observed relative to the vehicle group (Figure 2(A–D)). Golgi staining showed that PTXH significantly reduced the dendrite spine density (Figure 2(E,F). Moreover, PSD-95, a pivotal postsynaptic scaffolding protein in excitatory neurons that plays a key role in maintaining normal synaptic function, plasticity and signal transduction were decreased in the dendrites after PTX treatment (Figure 2(G,H)). BDNF is widely expressed in the central nervous system. It promotes the survival of nerve cells and increases synaptic plasticity and neurogenesis. Therefore, BDNF expression levels were determined by ELISA. The results showed that the expression of BDNF in the hippocampal tissue of mice treated with PTX was decreased significantly (Figure 2(I)).

### RIP3/MLKL-mediated necroptosis occurred in the hippocampus

**Figure 3.**
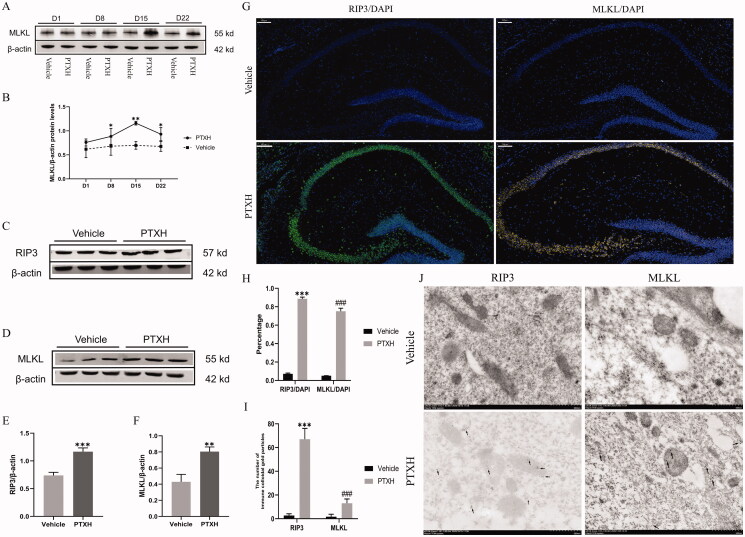
The expression of RIP3 and MLKL in the hippocampus of mice treated with PTX. (A) The expression of MLKL at different PTX treatment times. (B) The relative expression of MLKL as shown in A. (C, D, E, F) The expression of RIP3 and MLKL on the 15th day after treatment with PTX. (G, H) Immunofluorescence staining of RIP3 and MLKL (scale bar = 100 μm). (I, J) The deposition of immunocolloidal gold particles observed by high-power electron microscope (scale bar = 500 nm). The results are presented as the mean ± *SD*, *n* = 3. In the PTXH group, *p < 0.05 vs. vehicle, **p < 0.01 vs. vehicle, ***p < 0.001 vs. vehicle, ^###^*p*< 0.001 vs. vehicle.

The hippocampal tissues of mice were obtained at different time points after treatment with PTX. In order to observe whether necroptosis occurred in the hippocampus, the MLKL and RIP3 proteins, which form the key complexes in the process of necroptosis, were detected in western blot experiments. High expression of the MLKL protein in the hippocampus was first observed on day 8 and lasted until day 22, as compared with the vehicle group (Figure 3(A)). The peak value was reached on day 15 (Figure 3(B)). Unfortunately, no similar meaningful changes in the expression of RIP3 were observed. However, on day 15, a significant increase in the expression of both RIP3 and MLKL was observed, as compared with the vehicle group (Figure 3(C–F)). The expression of RIP3 and MLKL in the hippocampus was also observed by immunofluorescence staining. As shown in Figure 3(G,H), RIP3 and MLKL were highly expressed in the hippocampus of mice treated with PTXH, as compared with the vehicle group. The immunoelectron microscopy results showed that RIP3 and MLKL immune colloidal gold particles were highly expressed in the hippocampal cells of mice treated with PTXH (Figure 3(I,J)).

**Figure 4.**
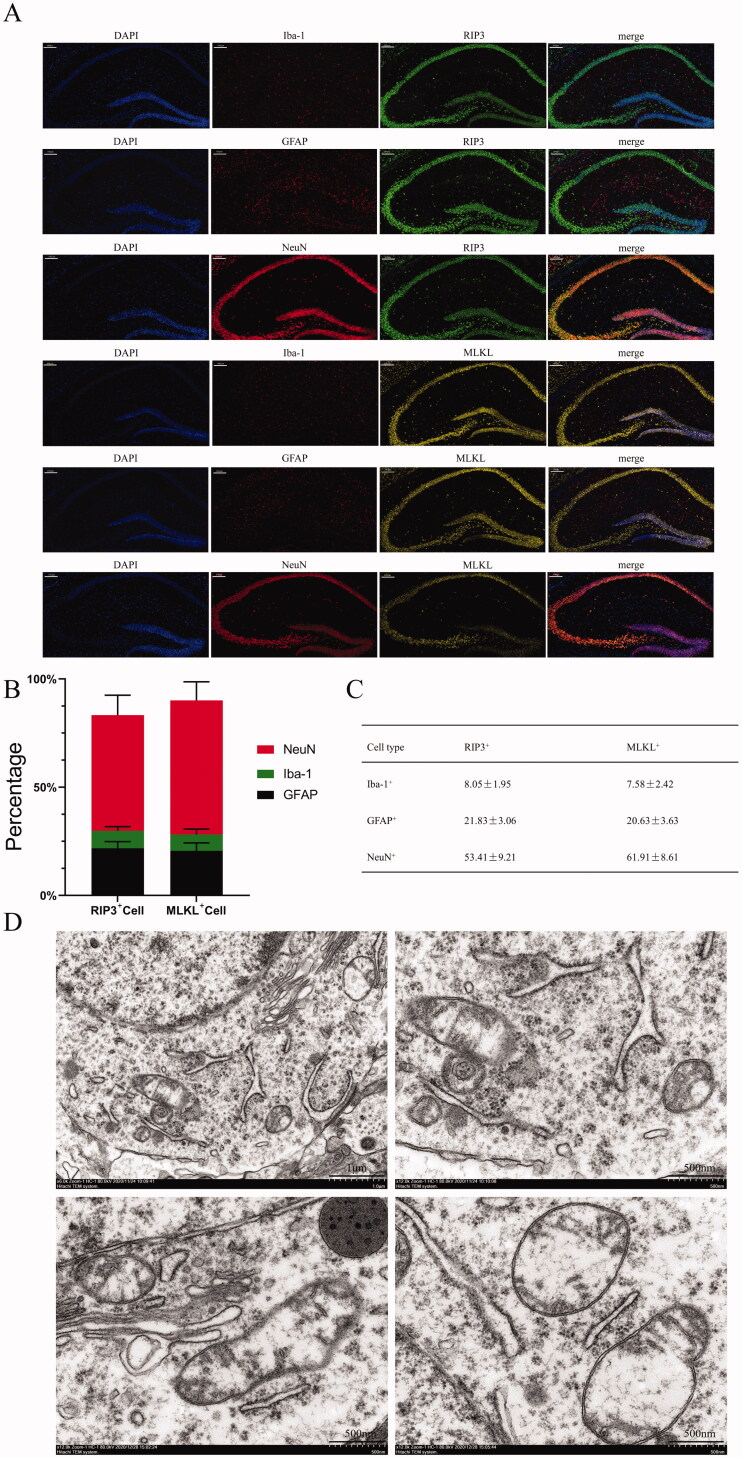
The expression of RIP3 and MLKL in different types of cells in the hippocampus. (A) Immunofluorescence double-staining of RIP3/MLKL and astrocytes/microglia/neurons (scale bar = 100 μm). (B, C) The proportions of different types of RIP3-positive and MLKL-positive cells. (D) Transmission electron microscopy scans of hippocampal neurons (scale bar = 1 μm or 500 nm). The results are presented as the mean ± *SD*, *n* = 3.

Then, two specific proteins were used as markers to observe the cell types that underwent necroptosis. The immunofluorescence staining results indicated that neurons were the main cells that underwent necroptosis, followed by astrocytes and microglia (Figure 4(A–C)). Moreover, the ultrastructural changes in necroptotic neurons were observed, including the dissolution of cytoplasm, the swelling of organelles, and the rupture of cell membranes (Figure 4(D)).

### PTX induced M1 polarisation of microglia in the hippocampus of mice

**Figure 5.**
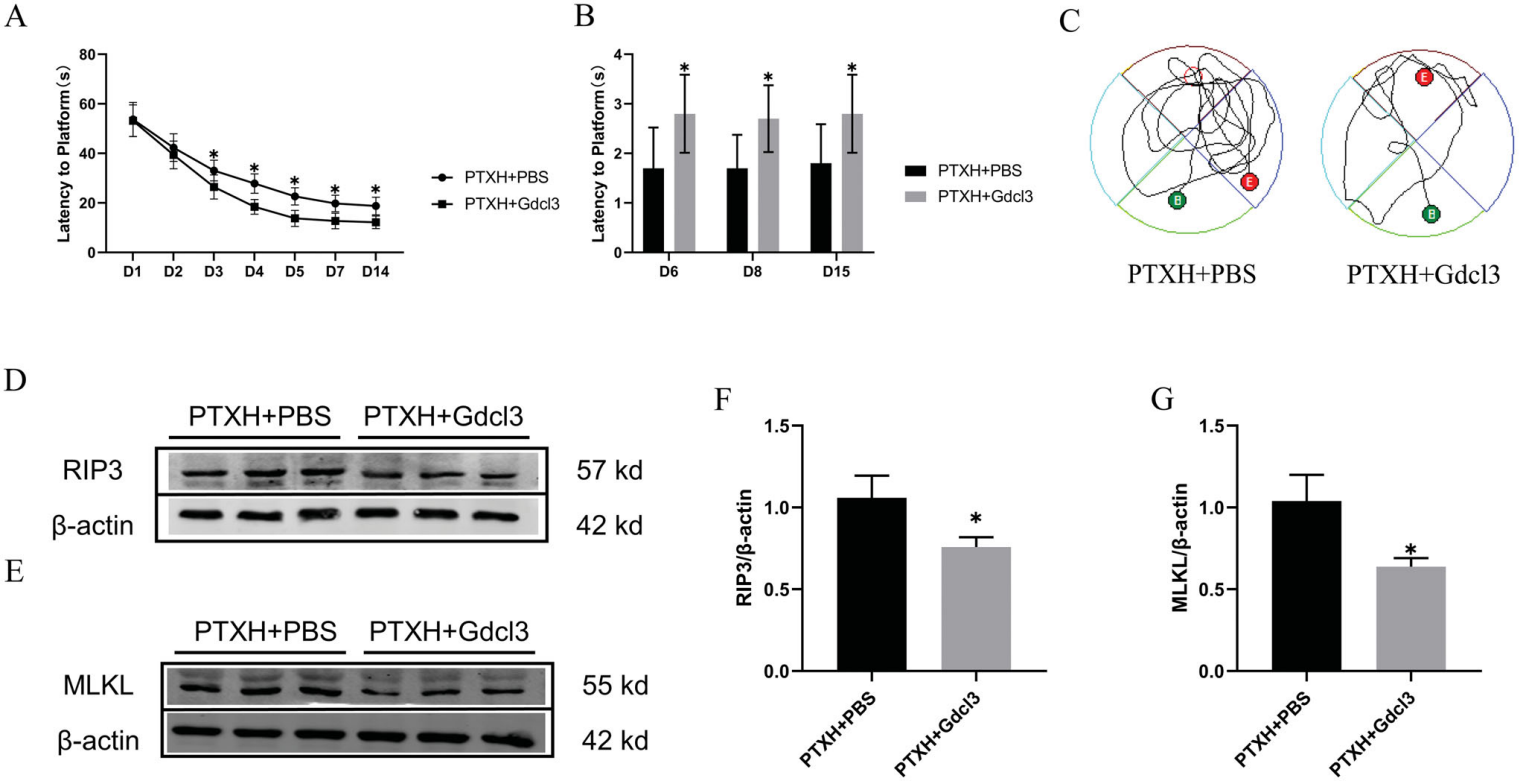
Effect of Gdcl3 on the cognitive impairment induced by PTX. (A) The escape latency(s). (B) The number of times passing the platform. (C) Movement trajectory on the 15th day. (D, E, F, G) The expression of the RIP3 and MLKL proteins. The results are presented as the mean ± *SD*, *n* = 10 or 3. In the PTXH + Gdcl3 group, *p < 0.05 vs. PTXH.

Microglia are intrinsic cells in the nervous system that play important roles in the neuroinflammatory response. The results of the behavioural test showed that Gdcl3, a scavenger of microglia, effectively reduced the neurotoxic effects of PTXH, which, in turn, rescued the spatial learning and memory impairments in mice (Figure 5(A–C)). This suggests that the neuroinflammatory response may be one of the mechanisms underlying PTX-induced cognitive impairment.

**Figure 6.**
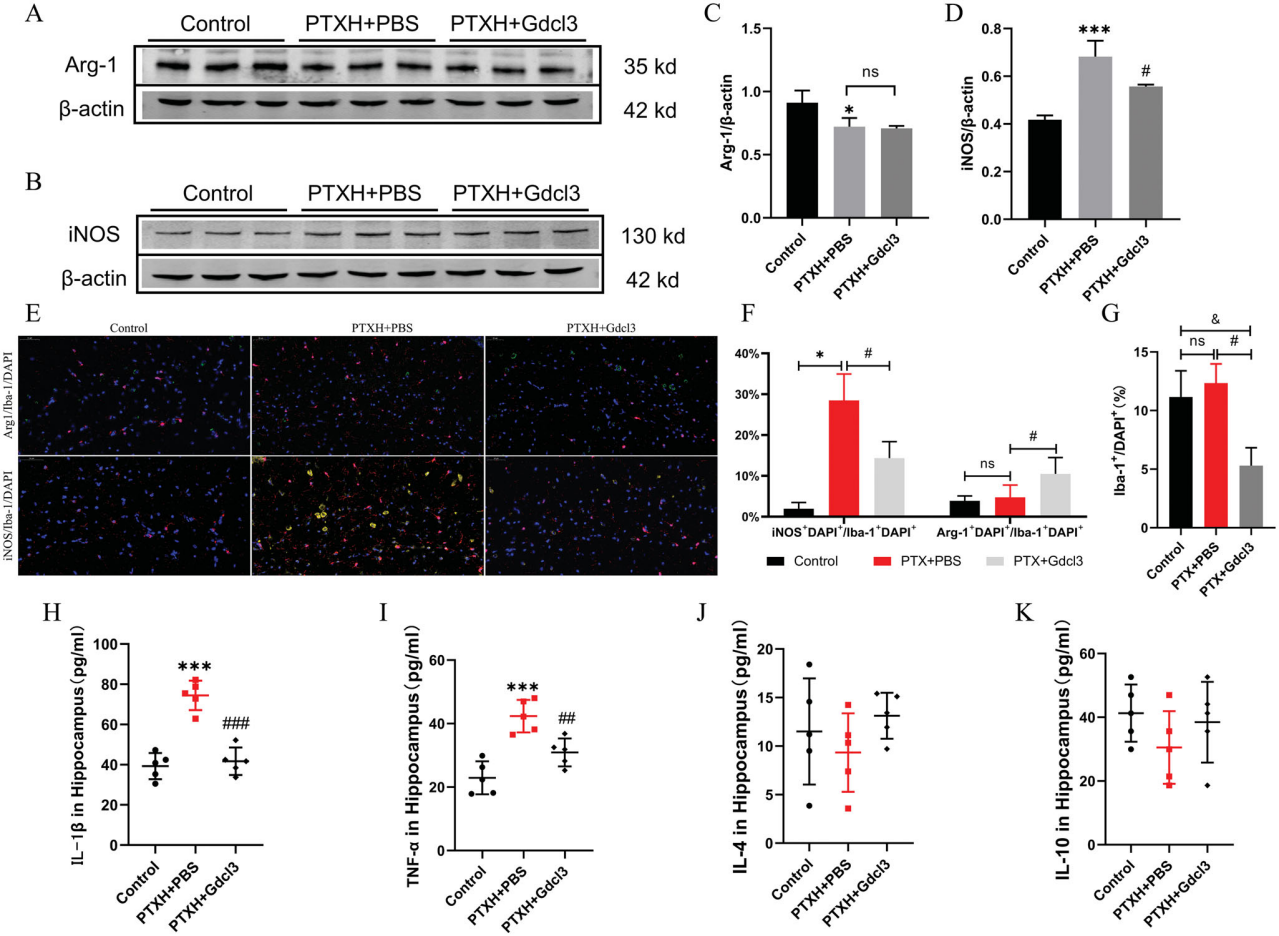
The polarization of microglia involved in PTX-induced cognitive impairment. (A, B, C, D) The expression of iNOS and Arg-1. (E) Polarization of microglia labelled with iNOS/Arg-1 and Iba-1 (scale bar = 50 μm). (F) The proportion of iNOS positive and Arg-1 positive cells to microglia. (G) The number of Iba-1 positive cells. (H, I, J, K) The concentration values of TNF-α, IL-1β, IL-4 and IL-10. The results are presented as the mean ± *SD*, *n* = 3 or 5. In the PTXH group, *p < 0.05 vs. vehicle, ***p < 0.001 vs. vehicle. In the PTXH + Gdcl3 group, ^#^*p* < 0.05 vs. PTXH, ^###^*p* < 0.001 vs. PTXH, ^&^*p* < 0.05 vs. vehicle.

As shown in Figure 5(D–G), we further verified that the removal of microglia is beneficial to reducing necroptotic cells; this was demonstrated by a significant decrease in the expression of RIP3 and MLKL in the PTXH + Gdcl3 group compared with the PTXH + PBS group. Then, the specific markers corresponding to the different types of microglia were comprehensively investigated. Western blot and immunofluorescence staining showed that PTX induced M1 polarisation of microglia, which was characterised by a significant increase in the expression of Arg1, accompanied by inhibition of the number of M2-type polarised microglia, while Gdcl3 effectively reduced the number of M1-type and M2-type polarised microglia (Figure 6(A–G)). The same phenomenon was also observed in the levels of inflammatory factors. Specifically, PTX induced a significant increase in the release of inflammatory factors such as TNF-α and IL-1 β, while the expression of these inflammatory factors decreased after the Gdcl3 intervention (Figure 6(H–K)). These results suggest that the M1 polarisation of microglia plays a key role in PTX-induced cognitive impairment.

## Discussion

The results of the present study indicated that PTX-induced cognitive impairment in adult mice was mediated by the dose and duration of PTX treatment. Moreover, PTX induced synaptic structural damage and significantly decreased synaptic plasticity in the hippocampi of mice. Further studies confirmed that RIP3/MLKL-mediated necroptosis occurred in the hippocampi of cognitively impaired mice; this peaked two weeks after the first PTX treatment. The necroptotic cells were mainly neurons, followed by astrocytes and microglia. Furthermore, M1 polarisation and the inflammatory response of microglia in the hippocampus were found to be involved in the occurrence of PTX-induced cognitive impairment in mice. Pre-treatment with Gdcl3, a microglial scavenger, effectively alleviated the PTX-induced cognitive impairment. These findings suggest that neuronal necroptosis, synaptic dysfunction and M1 polarisation of microglia are important causes of the cognitive impairment induced by PTX.

Animal models are the most common experimental approaches used to study disorders of cognition, such as AD, PD and ALS. However, PTX-induced cognitive impairment has received little research attention, until recently. Most studies of PTX, to date, have revealed only mild neurological damage, including anxiety or depression. In this study, the dosage of PTX was increased and the resulting damage to cognitive function was observed over a longer period of time. In this study, cognitive impairment induced by PTX was observed in C57bl/6n mice, and this model is very stable. The results revealed positive correlations between the concentrations and durations of PTX and the resulting cognitive impairment. However, at present, there is insufficient behavioural evidence to support this conclusion. Thus, in the future, we plan to use this model to conduct more detailed experiments to clarify the relationships between cognitive impairment and both PTX concentration and duration.

PTX-induced cognitive impairment is a severe neurological complication, and the symptoms last for months to years (Schagen et al. 1999; Klemp et al. 2018). Scholars have shown that the neurotoxicity of PTX underlies the cognitive impairment induced by this drug. The hippocampus, a brain region critical to cognition, is most vulnerable to PTX-induced neurotoxicity (Finke et al. 2017). It has been confirmed that PTX controls the proliferation and division of tumour cells by affecting the stability of tubulin, and the changes in the stability of tubulin directly affect the synaptic plasticity and memory formation of neurons (Atarod et al. 2015; Panoz-Brown et al. 2017; You et al. 2018). It is possible that the occurrence of neurons and the decrease in the number of cells may be one of the mechanisms underlying the cognitive impairment induced by PTX.

As a special form of cell death, necroptosis has been shown to be widely involved in the occurrence of neurodegenerative diseases. Here, it was demonstrated that necroptosis is involved in the process of cognitive impairment induced by PTX. Most necroptotic cells were hippocampal neurons, with some astrocytes and microglia also exhibiting necroptosis. The destruction of the hippocampal structure caused by the death of hippocampal cells may be the pathophysiological basis of cognitive impairment. This finding provides insight for further exploration of the mechanism and opens up the possibility of new necroptosis-targeted therapeutics for PTX-induced cognitive impairment.

The results of this study indicated that different cells exhibit different degrees of necroptosis. Oxidative stress is an important mechanism involved in necroptosis (Linkermann and Green 2014). Therefore, the degree of tolerance of different kinds of cells to oxidation determines the number of cell deaths. Previous studies have shown that microglia have the strongest tolerance to oxidative stress, while astrocytes rank second and neurons are the most sensitive to ROS (Bolaños et al. 1997). These findings are consistent with the necroptosis findings of the current study and may explain the different degrees of necroptosis in different cells in the current study. However, this is just speculation based on the experimental results and requires confirmation in future in-depth experiments.

Evidence suggests that astrocytes may regulate the synaptic connectivity of neural networks by supporting the metabolism of neurotransmitters, synaptic formation, synaptic elimination and synaptic plasticity, thus contributing to cognitive function, including learning, memory, emotion and behaviour (Augusto-Oliveira et al. 2020). In the current experiment, PTX induced damage to synaptic structure and function and caused necroptosis of astrocytes. These results suggest that the cognitive impairment induced by PTX may be closely related to over-activation/necroptosis of astrocytes. Our previous research demonstrated that PTX can increase the metabolic activity of mitochondria and enhance the oxidative stress response of cells (Li et al. 2019). These changes not only promote the occurrence of the neuroinflammatory response and neuronal degeneration, but also affect the transmission of synaptic information between neurons in the hippocampus. BDNF is a neurotrophic factor regulated by the level of PGC-1 α/FNDC5. It is an important component that regulates synaptic plasticity and affects cognition, learning and memory. Experiments have demonstrated an inhibitory effect of PTX on BDNF, which ultimately changes synaptic plasticity and leads to cognitive impairment. Studies of the development of neurodegenerative diseases have demonstrated that change to synaptic structure and decreased synaptic plasticity also play key roles in the process of cognitive decline.

Microglia are innate immune cells of the nervous system that participate in the occurrence and development of a variety of diseases. Most of the time, the microglia in the brain act as housekeepers, constantly monitoring changes in the neural microenvironment. Under different environmental stimuli, microglia can polarise from the resting state of M0 to the M1 or M2 state. The main function of M1 is to release inflammatory factors and promote the inflammatory response, while M2 can promote tissue microenvironment repair and eliminate the inflammatory response. M1 polarisation and neuroinflammation of microglia are also associated with aging-related cognitive decline and the progression of neurodegenerative diseases, including AD (Suenaga et al. 2015; Xiong et al. 2016; Kim and Bae 2017; Li and Barres 2018; Park et al. 2021). The results of this study confirmed that microglial M1 polarisation and the neuroinflammatory response are involved in the cognitive impairment induced by PTX. M1-type microglia can release a large number of inflammatory factors such as TNF-α and IL-1 β, resulting in strong neurotoxicity. At the same time, activated M1 microglia can participate in excessive synaptic pruning of neurons through phagocytosis, which leads to synaptic plasticity disorder and affects cognitive ability. After the use of GdCl3 to remove microglia, the above damage was reversed, and the cognitive status of mice was significantly improved.

## Conclusions

This study established a PTX-induced cognitive impairment animal model, and a variety of experimental methods confirmed that PTX-induced cognitive impairment is closely related to necroptosis, synaptic dysfunction and M1 polarisation of microglia. Pre-treatment with Gdcl3 inhibited microglia, effectively reduced the release of inflammatory factors, and improved the cognitive function of mice damaged by PTX. These findings suggest important mechanisms underlying PTX-induced cognitive impairment and pave the way for the identification of new methods and treatment approaches for solving PTX-induced impairments in clinical practice.

## References

[CIT0001] Atarod D, Eskandari-Sedighi G, Pazhoohi F, Karimian S, Khajeloo M, Riazi G. 2015. Microtubule dynamicity is more important than stability in memory formation: an *in vivo* study. J Mol Neurosci. 56(2):313–319. 25740015 10.1007/s12031-015-0535-4

[CIT0002] Augusto-Oliveira M, Arrifano G, Takeda P, Lopes-Araújo A, Santos-Sacramento L, Anthony D, Verkhratsky A, Crespo-Lopez M. 2020. Astroglia-specific contributions to the regulation of synapses, cognition and behaviour. Neurosci Biobehav Rev. 118:331–357. 32768488 10.1016/j.neubiorev.2020.07.039

[CIT0003] Bolaños J, Almeida A, Stewart V, Peuchen S, Land J, Clark J, Heales S. 1997. Nitric oxide-mediated mitochondrial damage in the brain: mechanisms and implications for neurodegenerative diseases. J Neurochem. 68(6):2227–2240. 9166714 10.1046/j.1471-4159.1997.68062227.x

[CIT0004] Boyette-Davis J, Fuchs P. 2009. Differential effects of paclitaxel treatment on cognitive functioning and mechanical sensitivity. Neurosci Lett. 453(3):170–174. 19429028 10.1016/j.neulet.2009.02.031

[CIT0005] Finke C, Prüss H, Heine J, Reuter S, Kopp U, Wegner F, Then Bergh F, Koch S, Jansen O, Münte T, et al. 2017. Evaluation of cognitive deficits and structural hippocampal damage in encephalitis with leucinerich, glioma-inactivated 1 antibodies. JAMA Neurol. 74:50–59. 27893017 10.1001/jamaneurol.2016.4226

[CIT0006] Fischer R, Maier O. 2015. Interrelation of oxidative stress and inflammation in neurodegenerative disease: role of TNF. Oxidative Med Cell Longevity. 2015:1–18. 10.1155/2015/610813 PMC4365363 25834699

[CIT0007] Han S, Park J, Mook-Jung I. 2016. Amyloid β-interacting partners in Alzheimer’s disease: from accomplices to possible therapeutic targets. Prog Neurobiol. 137:17–38. 26721621 10.1016/j.pneurobio.2015.12.004

[CIT0008] He S, Wang L, Miao L, Wang T, Du F, Zhao L, Wang X. 2009. Receptor interacting protein kinase-3 determines cellular necrotic response to TNF-alpha. Cell. 137(6):1100–1111. 19524512 10.1016/j.cell.2009.05.021

[CIT0009] Jang S, Chung H. 2016. Emerging link between Alzheimer’s disease and homeostatic synaptic plasticity. Neural Plast. 2016:7969272. 27019755 10.1155/2016/7969272 PMC4785275

[CIT0010] Karavelioglu E, Gonul Y, Aksit H, Boyaci M, Karademir M, Simsek N, Guven M, Atalay T, Rakip U. 2016. Cabazitaxel causes a dose-dependent central nervous system toxicity in rats. J Neurol Sci. 360:66–71. 26723976 10.1016/j.jns.2015.11.033

[CIT0011] Kim J, Bae H. 2017. Spontaneous intracerebral hemorrhage: management. J Stroke. 19(1):28–39. 28178413 10.5853/jos.2016.01935 PMC5307946

[CIT0012] Klemp J, Myers J, Fabian C, Kimler B, Khan Q, Sereika S, Stanton A. 2018. Cognitive functioning and quality of life following chemotherapy in pre- and peri-menopausal women with breast cancer. Support Care Cancer. 26(2):575–583. 28849337 10.1007/s00520-017-3869-3 PMC5754254

[CIT0013] Layé S, Gheusi G, Cremona S, Combe C, Kelley K, Dantzer R, Parnet P. 2000. Endogenous brain IL-1 mediates LPS-induced anorexia and hypothalamic cytokine expression. Am J Physiol Regul Integr Comp Physiol. 279(1):R93–98. 10896869 10.1152/ajpregu.2000.279.1.R93

[CIT0014] Li Q, Barres B. 2018. Microglia and macrophages in brain homeostasis and disease. Nat Rev Immunol. 18(4):225–242. 29151590 10.1038/nri.2017.125

[CIT0015] Li X, Yang S, Wang L, Liu P, Zhao S, Li H, Jiang Y, Guo Y, Wang X. 2019. Resveratrol inhibits paclitaxel-induced neuropathic pain by the activation of PI3K/Akt and SIRT1/PGC1α pathway. JPR. ume 12:879–890. 10.2147/JPR.S185873 PMC6404678 30881098

[CIT0016] Li Z, Zhao S, Zhang H, Liu P, Liu F, Guo Y, Wang X. 2018. Proinflammatory factors mediate paclitaxel-induced impairment of learning and memory. Mediators Inflamm. 2018:3941840. 29681766 10.1155/2018/3941840 PMC5842689

[CIT0017] Linkermann A, Green D. 2014. Necroptosis. N Engl J Med. 370(5):455–465. 24476434 10.1056/NEJMra1310050 PMC4035222

[CIT0018] Malenka R, Bear M. 2004. LTP and LTD: an embarrassment of riches. Neuron. 44(1):5–21. 15450156 10.1016/j.neuron.2004.09.012

[CIT0019] Mifflin L, Ofengeim D, Yuan J. 2020. Receptor-interacting protein kinase 1 (RIPK1) as a therapeutic target. Nat Rev Drug Discov. 19(8):553–571. 32669658 10.1038/s41573-020-0071-y PMC7362612

[CIT0020] Neves G, Cooke S, Bliss T. 2008. Synaptic plasticity, memory and the hippocampus: a neural network approach to causality. Nat Rev Neurosci. 9(1):65–75. 18094707 10.1038/nrn2303

[CIT0021] Panoz-Brown D, Carey L, Smith A, Gentry M, Sluka C, Corbin H, Wu J, Hohmann A, Crystal J. 2017. The chemotherapeutic agent paclitaxel selectively impairs reversal learning while sparing prior learning, new learning and episodic memory. Neurobiol Learn Mem. 144:259–270. 28811227 10.1016/j.nlm.2017.08.001 PMC5621653

[CIT0022] Park J, Ha H, Chung E, Baek S, Cho Y, Kim H, Han J, Sul J, Lee J, Kim E, et al. 2021. *O*-GlcNAcylation ameliorates the pathological manifestations of Alzheimer’s disease by inhibiting necroptosis. Sci Adv. 7:1–17. 10.1126/sciadv.abd3207 PMC7806231 33523877

[CIT0023] Re D, Le Verche V, Yu C, Amoroso M, Politi K, Phani S, Ikiz B, Hoffmann L, Koolen M, Nagata T, et al. 2014. Necroptosis drives motor neuron death in models of both sporadic and familial ALS. Neuron. 81(5):1001–1008. 24508385 10.1016/j.neuron.2014.01.011 PMC3951532

[CIT0024] Schagen S, van Dam F, Muller M, Boogerd W, Lindeboom J, Bruning P. 1999. Cognitive deficits after postoperative adjuvant chemotherapy for breast carcinoma. Cancer. 85(3):640–650. 10091737 10.1002/(sici)1097-0142(19990201)85:3<640::aid-cncr14>3.0.co;2-g

[CIT0025] Shen J, Li Y, Qu C, Xu L, Sun H, Zhang J. 2019. The enriched environment ameliorates chronic unpredictable mild stress-induced depressive-like behaviors and cognitive impairment by activating the SIRT1/miR-134 signaling pathway in hippocampus. J Affect Disord. 248:81–90. 30716615 10.1016/j.jad.2019.01.031

[CIT0026] Suenaga J, Hu X, Pu H, Shi Y, Hassan S, Xu M, Leak R, Stetler R, Gao Y, Chen J. 2015. White matter injury and microglia/macrophage polarization are strongly linked with age-related long-term deficits in neurological function after stroke. Exp Neurol. 272:109–119. 25836044 10.1016/j.expneurol.2015.03.021 PMC4591088

[CIT0027] Tannock I, Ahles T, Ganz P, Van Dam F. 2004. Cognitive impairment associated with chemotherapy for cancer: report of a workshop. J Clin Oncol. 22(11):2233–2239. 15169812 10.1200/JCO.2004.08.094

[CIT0028] Xiong X, Liu L, Yang Q. 2016. Functions and mechanisms of microglia/macrophages in neuroinflammation and neurogenesis after stroke. Prog Neurobiol. 142:23–44. 27166859 10.1016/j.pneurobio.2016.05.001

[CIT0029] You Z, Zhang S, Shen S, Yang J, Ding W, Yang L, Lim G, Doheny J, Tate S, Chen L, et al. 2018. Cognitive impairment in a rat model of neuropathic pain: role of hippocampal microtubule stability. Pain. 159(8):1518–1528. 29613911 10.1097/j.pain.0000000000001233 PMC6053326

